# Report of the Signal Transduction Society Meeting 2018—Signaling: From Past to Future

**DOI:** 10.3390/ijms20010227

**Published:** 2019-01-08

**Authors:** Bastian Schirmer, Klaudia Giehl, Katharina F. Kubatzky

**Affiliations:** 1 Institut für Pharmakologie, Medizinische Hochschule Hannover, Carl-Neuberg-Str. 1, 30625 Hannover, Germany; schirmer.bastian@mh-hannover.de; 2 Signaltransduktion zellulärer Motilität, Innere Medizin V, Justus-Liebig-Universität Giessen, Aulweg 128, 35392 Giessen, Germany; klaudia.giehl@inneremed.jlug.de; 3 Zentrum für Infektiologie, Medizinische Mikrobiologie und Hygiene, Universitätsklinikum Heidelberg, Im Neuenheimer Feld 324, 69120 Heidelberg, Germany

**Keywords:** signal transduction, STS, conference report, receptor signaling, neuronal signaling, infection and inflammation, growth factors, cytokines, immune cell signaling, cancer research

## Abstract

The annual meeting “Signal Transduction—Receptors, Mediators, and Genes” of the Signal Transduction Society (STS) is an interdisciplinary conference open to all scientists sharing the common interest in elucidating signaling pathways in physiological or pathological processes in humans, animals, plants, fungi, prokaryotes, and protists. On the occasion of the 20th anniversary of the STS, the 22nd joint meeting took place in Weimar from 5–7 November 2018. With the focus topic “Signaling: From Past to Future” the evolution of the multifaceted research concerning signal transduction since foundation of the society was highlighted. Invited keynote speakers introduced the respective workshop topics and were followed by numerous speakers selected from the submitted abstracts. All presentations were lively discussed during the workshops. Here, we provide a concise summary of the various workshops and further aspects of the scientific program.

## 1. Introduction

The 22nd Joint Meeting “Signal Transduction—Receptors, Mediators and Genes” was held in Weimar, Germany, from 5 to 7 November 2018 [1]. As in past years, the Signal Transduction Society (STS), the signaling study groups of the German Societies of Pharmacology (DGP), Cell Biology (DGZ), Physiology (DPG), Biochemistry and Molecular Biology (GBM), and Immunology (DGfI) had jointly organized the meeting. Further scientific and financial support was provided by the collaborative research center (SFB) 854 “Molecular Organization of Cellular Communication in the Immune System” (B. Schraven, Magdeburg, DE) and the study group “Infection Immunology” of the DGfI and the German Society for Hygiene and Medical Microbiology (DGHM). The support of young researchers has always been a key element of STS meetings and again numerous next-generation scientists presented their work with posters and oral presentations. In order to integrate the young scientists even more, junior groups of the DGP, DPG, and DGfI for the first time contributed to the STS meeting by organizing distinctive workshops. In view of the 20th anniversary of the Signal Transduction Society in 2018, the focus topic of the meeting “Signaling: From Past to Future” had been chosen to emphasize the evolution of the multifaceted research concerning signal transduction since the foundation of the society.

## 2. Meeting Overview

### 2.1. Keynote Lectures

According to the focus theme “Signaling: From Past to Future” each workshop was introduced by an expert in the respective field, who gave an overview of how his or her research field has evolved. 

The first workshop “Growth factors, Cytokines and their Receptors” was opened by Manuela Baccarini (Vienna, AT), who presented her research on the non-canonical functions of ERK pathway components. Beyond their well-known functions as kinases of the ERK signaling module, RAF and MEK have several kinase-independent effects. These effects are mostly based on the ability of RAF/MEK to physically interact with other signal transduction proteins. Furthermore, the Baccarini group provided evidence for distinct in vivo functions of RAF and MEK, thereby laying ground for new possibilities in targeted anti-cancer therapy [2,3].

Manolis Pasparakis (Cologne, DE) opened the workshop on “Differentiation, Stress and Death” by presenting his work on receptor-interacting protein kinase (RIPK) signaling in cell death and its critical role in immune responses. There is a complex interplay between IKK/NF-kB and RIPK1 signaling modules influencing the balance between tissue homeostasis and inflammation in epithelial barrier tissues. In this context, IKK subunits can inhibit RIPK1 activity both NF-kB-dependently and –independently. Moreover, since it could be shown that RIPK1 and RIPK3 have kinase-dependent and kinase-independent effects, the Pasparakis group set out to elucidate the processes regulating these complex interplays and their in vivo significances. As an example, the deubiquitinating protein A20 targets RIPK3, MyD88, and NLRP-3 signaling molecules of the NF-kB pathway and absence of functional A20 in mice leads to severe multifocal inflammation, whereas specific ablation of A20 in myeloid cells leads to a spontaneous onset of destructive polyarthritis [4,5,6].

The introductory keynote presentation for the workshop on “Tumor Cell Biology” was held by Martin Eilers (Würzburg, DE), who gave an informative and concise overview of the function of the transcription factor N-Myc in human neuroblastoma. Myc proteins are bound to virtually any active promotor in the human genome and can be found dysregulated not only in neuroblastoma, but in most human cancers. In this context, the Eilers group found evidence for a regulatory function of the kinase Aurora-A, which is expressed cell cycle-dependently and prevents N-Myc degradation by the ubiquitin ligase Fbxw7. During S Phase Aurora-A competes with RAD21 and TFIIIC2 for N-Myc binding, thereby preventing promotor escape and pause release of RNA polymerase II, which is mediated by the N-Myc/TFIIIC2/RAD21 complex. Thus, pharmacological inhibition of Aurora-A has the potential to inhibit proliferation of neuroblastoma cells overexpressing N-Myc, as demonstrated by increased tumor regression and improved survival in a mouse model of neuroblastoma [7,8,9]. 

Björn Lillemeier (La Jolla, CA, USA) presented his work on the effects of membrane dynamics of signaling molecules on T cell responses in the workshop “Immune Cell Signaling II” (The first workshop on immune cell signaling had been organized jointly with the junior group of the DGfI and mentioned below). He showed a plethora of exciting data on the organization of signaling molecules in nanometer scale plasma membrane islands, such as the T cell receptor (TCR) nanocluster and the nanoclusters containing the Zap70 substrate LAT. After activation of the T cells, the protein islands concatenate into microclusters. TCR signals are then relayed to LAT islands via translocation of Zap70 between the nanodomains in a ‘catch-and-release’ mechanism. For high-resolution imaging of the membrane dynamics, the Lillemeier group has developed sophisticated approaches combining single-molecule super-resolution and dynamic fluorescence microscopy with traditional biochemical and immunological techniques [10,11,12]. 

All of these keynote lectures were followed by a number of short talks selected from the submitted abstracts. As in the past, the stimulating blend of presentations given by young scientists, early career researchers and established scientists was an outstanding feature of the meeting.

### 2.2. Workshops Co-Organized by Young Scientists

The DGP junior group “Forum Junge Wissenschaft” organized the workshop “G Proteins and G Protein coupled receptors” with 5 early career researchers as speakers. Christoph Klenk (Zürich, CH) gave insights into his research concerning directed evolution of the human parathyroid hormone 1 receptor (PTH1R), which made it possible to obtain more thermally stable variants and subsequently a valid crystal structure of PTH1R [13]. The focus on structural studies was shared by the following speaker Michael Kauk (Jena, DE), who presented his work on muscarinic acetylcholine receptors (mAChR). He used FRET- and BRET-based analyses as well as different derivatives of the mAChR ligand iperoxo to investigate a possible gatekeeper function of an aromatic lid structure within the mAChR, which might convey subtype selectivity for certain ligands [14,15]. Andrea Kliewer (Jena, DE) introduced the concept of G protein bias at µ-opioid receptors (MOR) to the audience, where differential phosphorylation of MOR by GPCR kinases leads to altered G protein signaling as demonstrated in vivo with a series of different phosphorylation-deficient mice [16,17]. In contrast to MOR, adhesion GPCRs, which are the research interest of Simone Prömel (Leipzig, DE), are considerably less well studied. During her talk, S. Prömel portrayed the peculiar dual signal transduction of the adhesion GPCR latrophilin with a *cis* mode driven by classical G Protein signaling and a *trans* mode conveyed by the N-terminus of latrophilin in a non-cell-autonomous manner [18,19]. The session was concluded by Stefania Mariggiò (Naples, IT), who presented her research on the role of the lysophosphatidylinositol/GPR55 axis in regulating osteoclast differentiation. Via phage display, she was able to generate peptide binders of GPR55, which inhibited RANKL-induced osteoclastic differentiation of RAW264.7 cells and could represent a new therapeutic strategy in managing bone diseases with GPR55 dysregulation [20].

The “Young Physiologists” group of the DPG organized the workshop “Neuronal Signaling and Ion Channels” with 5 speakers recruited from their junior group. The session was opened by Jan-Philipp Machtens (Jülich, DE), who introduced the complex field of neuronal signaling and ion channels to the audience. To that end, he presented his work on excitatory amino acid transporters (EAAT), which he had analyzed using e.g., patch clamp techniques and computational electrophysiologic modelling [21,22]. Maddalena Comini (Oxford, GB) focused on the chloride/proton exchangers ClC-3 and ClC-5, which she could demonstrate to be crucial during the priming phase of exocytosis of large dense core vesicles in chromaffin cells. Jan Meents (Aachen, DE) gave insight into his work on the voltage-gated sodium channel Na_v_1.7, which is dysfunctional in patients with the chronic pain syndrome erythromelalgia. Using induced pluripotent stem cells, which were differentiated into sensory nociceptors, he could establish a model system as a tool for development of new analgesics [23,24]. The session was completed by Gustavo Chaves (Nürnberg, DE), who shared his data on the selectivity and zinc inhibition of the voltage-gated proton channel H_v_1 with the audience. Applying pH titration and targeted mutation of the channel, he identified amino acid residues which are crucial for proton selectivity and zinc binding [25,26].

The “Young Immunologists” group of the DGfI participated in the organization of the workshop “Immune Cell Signaling I”, where they had chosen Martin Väth (Würzburg, DE) as keynote speaker. He spoke about his extensive analysis of the stromal interaction molecule (STIM) 1 and STIM2 in the context of the effector differentiation of regulatory T (Treg) cells. STIM1/2 mediates the first wave of cytosolic calcium increase during store-operated calcium entry (SOCE) signaling in Treg cells, which is known to control the thymic development of Foxp3^+^ Treg cells. Väth showed that ablation of STIM1/2 in mature Foxp3^+^ Treg cells significantly impairs the further differentiation into follicular Treg (Tfr) cells and tissue-resident Treg cells. In vivo, mice with SOCE deficient Treg cells exhibited a severe inflammatory phenotype with a variety of autoantibodies and fatal inflammation in multiple solid organs underlining the critical role of STIM1/2 during differentiation of effector Treg cells [27,28]. The keynote lecture was followed by 4 short talks, again selected from the submitted abstracts.

### 2.3. Fostering of Early Career Researchers

In 2018, the STS grant committee chose 9 master, MD, or PhD students to receive travel grants of 250 € each to allow their meeting attendance. One of the travel grants was sponsored by Biomol, the Silver Sponsor of the meeting. As part of the well-known “one minute—one transparency” session, each poster-presenting scientist had the opportunity to introduce their research. Subsequently, the audience had the possibility to meet the presenting scientists at their posters in a casual atmosphere, discuss their data, and establish contacts. Overall, 7 poster prizes to a total value of 750 € were awarded to the most excellent posters, which had been selected by the chair people of the various workshops at the meeting.

The STS Science Award is meant to honor outstanding research by a post doc or junior principal investigator of the STS. It was first introduced at the annual STS Meeting in 2005 and ever since it has become a regular element of all following STS Meetings. This year, Dirk Brenner (Luxemburg, LU) received the STS Science Award for his comprehensive research on the glutathione-dependent regulation of metabolic responses in B and T cells. The award, with a total sum of 1500 €, was donated by the STS and OMNI Life Science GmbH. 

### 2.4. GBM Innovation Award

Annually on the occasion of the STS Joint Meeting, the GBM announces the GBM Innovation Award for Young Scientists to the value of 500 €. The Award is meant to honor young scientists working on an innovative and interesting method in the field of signal transduction. An award jury consisting of the speakers of the GBM-Study Groups “Biochemical Pharmacology and Toxicology” and “Receptors and Signal Transduction” selects appropriate candidates from the applications. In 2018, Theresia Gutmann (Dresden, DE) received the award for developing an in vitro membrane-mimetic system by embedding purified full-length insulin receptors in so-called lipid nanodiscs, and directly visualizing the conformational change induced by insulin. This minimal in vitro system is a valuable technique for addressing signaling mechanisms of the insulin receptor or other receptor tyrosine kinases, respectively.

## 3. The STS Honorary Medal Award

The STS Honorary Medal was introduced in 2010 in order to honor outstanding scientists in the field of signal transduction. After a laudation for the awardee and the festive presentation of the medal, the laureate concludes the award session with the “Honorary Medal Lecture”. Previous winners of the award are Anthony Pawson, Tony Hunter, Carl-Henrik Heldin, Klaus Rajewsky, Jules Hoffmann, Mina Bissell, Tak Wah Mak, and Michael Reth. Since 2017, the medal is awarded by the STS in collaboration with the International Journal of Molecular Sciences (IJMS).

In 2018, Prof. Dr. Karen Vousden (London, GB) received the STS Honorary Medal Award for her lifetime contributions on the functions and regulation of the tumor suppressor p53. Not only has Prof. Vousden discovered MDM2 as a decisive regulator of p53 expression, but she has also uncovered how p53 induces cell death and how it regulates cell metabolism [29,30,31]. Importantly, her basic research on p53 and its targets has led to the development of novel therapeutic approaches targeting cancer [32,33]. Her work has significantly contributed to our current understanding of p53 as ‘the guardian of the genome’ and of its role in tumor development. Karen Vousden inspired, supported and guided generations of students and fellows. The laudatory speech was given by Volker Dötsch (Frankfurt am Main, DE), who, focusing his research on the p53-homolog p63, has been collaborating with Prof. Vousden for many years. In her Honorary Medal Lecture, Prof. Vousden covered the manifold aspects of p53 not only as central modulator of cell survival and death, but as regulator of the cell’s oxidative state, too. The lecture was highly appreciated by the audience and followed by a lively and inspiring discussion.

## 4. Final Remarks

The preparations of the 23rd STS Meeting have already started. Again, the meeting will take place at the Leonardo Hotel in Weimar from 4 November to 6 November 2019. Further details and regular updates on the schedule and contents of the meeting can be found at https://www.sigtrans.de and on the STS Facebook account.
